# The Changing Face of Water: A Dynamic Reflection of Antibiotic Resistance Across Landscapes

**DOI:** 10.3389/fmicb.2018.01894

**Published:** 2018-09-06

**Authors:** Claire E. Sanderson, J. Tyler Fox, Eric R. Dougherty, Andrew D. S. Cameron, Kathleen A. Alexander

**Affiliations:** ^1^Department of Fish and Wildlife Conservation, Virginia Tech, Blacksburg, VA, United States; ^2^Center for African Resources: Animals, Communities and Land Use, Kasane, Botswana; ^3^Department of Environmental Science, Policy, and Management, University of California at Berkeley, Berkeley, CA, United States; ^4^Institute for Microbial Systems and Society, Faculty of Science, University of Regina, Regina, SK, Canada; ^5^Department of Biology, University of Regina, Regina, SK, Canada

**Keywords:** antibiotic resistance, water quality, *Escherichia coli*, Botswana, dryland system

## Abstract

Little is known about the role of surface water in the propagation of antibiotic resistance (AR), or the relationship between AR and water quality declines. While healthcare and agricultural sectors are considered the main contributors to AR dissemination, few studies have been conducted in their absence. Using linear models and Bayesian kriging, we evaluate AR among *Escherichia coli* water isolates collected bimonthly from the Chobe River in Northern Botswana (*n* = 1997, *n* = 414 water samples; July 2011–May 2012) in relation to water quality dynamics (*E. coli*, fecal coliform, and total suspended solids), land use, season, and AR in wildlife and humans within this system. No commercial agricultural or large medical facilities exist within this region. Here, we identify widespread AR in surface water, with land use and season significant predicators of AR levels. Mean AR was significantly higher in the wet season than the dry season (*p* = 0.003), and highest in the urban landscape (2.15, *SD* = 0.098) than the protected landscape (1.39, *SD* = 0.051). In-water *E. coli* concentrations were significantly positively associated with mean AR in the wet season (*p* < 0.001) but not in the dry season (*p* = 0.110), with TSS negatively associated with mean AR across seasons (*p* = 0.016 and *p* = 0.029), identifying temporal and spatial relationships between water quality variables and AR. Importantly, when human, water, and wildlife isolates were examined, similar AR profiles were identified (*p* < 0.001). Our results suggest that direct human inputs are sufficient for extensive dispersal of AR into the environment, with landscape features, season, and water quality variables influencing AR dynamics. Focused and expensive efforts to minimize pollution from agricultural sources, while important, may only provide incremental benefits to the management of AR across complex landscapes. Controlling direct human AR inputs into the environment remains a critical and pressing challenge.

## Introduction

Antibiotic resistance (AR) is recognized as a critical global health emergency, with expected increases in deaths worldwide from diseases that were once treatable (World Health Organization, 2014). Antibiotics are routinely used in human and veterinary medicine to treat or prevent infectious disease. They are also often used in agricultural production systems where they can suppress bacterial diseases as well as promote the growth of livestock and crops. However, decades of misuse and over-consumption of antibiotics have exerted enormous selective pressures on bacterial communities, driving the evolution of resistance (Davies, 2007; Zhang et al., 2009). The wide range of antibiotics and the facility with which bacteria evolve and acquire resistance genes results in AR profiles that can range from resistance to a single antibiotic to multidrug resistance. An increasing diversity of AR bacteria and genes have been observed in both clinical and non-clinical settings, including soil, insects, domestic and wild animals (Allen et al., 2010; Pesapane et al., 2013; Jobbins and Alexander, 2015), and even humans in remote locations (Bartoloni et al., 2009).

Across environments, surface water remains a critical connecting resource. AR bacteria can be transmitted into surface water directly from hospital and municipal wastewater effluents, or indirectly from fecal runoff and infiltration from agricultural and animal husbandry practices (Zhang et al., 2009; Pruden et al., 2012; Rizzo et al., 2013). High levels of AR are routinely found in freshwater systems around urban wastewater and agricultural effluent inflow points (Schwartz et al., 2003; Lima-Bittencourt et al., 2007; Knapp et al., 2012; Pruden et al., 2012; Czekalski et al., 2014; Hsu et al., 2015). Once AR bacteria enter waterways, they may be ingested by animals or humans, or may transfer resistance genes to other bacteria in the environment (Bouki et al., 2013). These processes can occur across complex river flood plain habitats, surface water, sediments and animal communities, but our understanding of these processes is critically limited. While the healthcare and agricultural sectors are often identified as the main contributors to AR bacteria dissemination (Van Den Bogaard and Stobberingh, 2000; World Health Organization, 2001), few studies have been conducted in populated regions where these enterprises are absent, potentially obscuring the role of direct human inputs in AR dissemination. The potential relationship between water quality declines [increases in fecal microbial communities and total suspended solids (TSS)] and AR dynamics is also unknown.

Using our study system in Northern Botswana, we evaluate the spatial and temporal dynamics of AR movement in the only permanent surface water in the region, the Chobe River, in relation to season, land use, water quality dynamics, and AR accumulation in associated human and wildlife populations. This human populated system provides an ideal location to evaluate these dynamics as commercial livestock production and large health facilities influences are absent.

## Methods

### Study site

**Figure 1:**
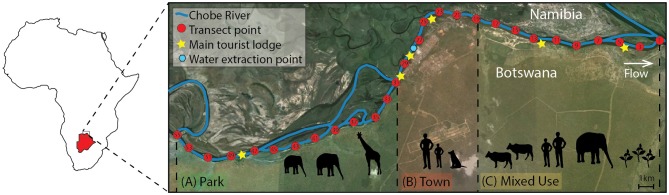
The study site is located in Northern Botswana (red map inset) in sub-Saharan Africa, and spans three different land use types (Town, Mixed Use, and Park, shown in red, yellow, and green, respectively). Transect points for water sample collection are noted in red and are located at 1 km intervals along the Chobe River. Tourist lodges (stars) and the water intake point in Town (blue hexagon) are noted.

This study was conducted in the Chobe District in Northern Botswana (Figure 1). The Chobe River is a dryland flood pulse system and one of only three permanent sources of surface water in Botswana. This river system supplies potable water to the resident populations living across nine villages spanning urban to rural landscapes within a biodiversity hot spot. Municipal water is piped directly from the Chobe River at a water plant located downstream from the Chobe National Park (Figure 1). The region experiences pronounced seasonal hydrologic variability with distinct wet (November-March) and dry seasons (April-October) with variable rainfall and periods of drought that appear to occur on a 10-year cycle (Alexander and Blackburn, 2013; Alexander et al., 2013; Fox and Alexander, 2015). In this system, rainfall and the flood pulse provide the dominant ecological drivers with the peak flood occurring at the beginning of the dry season (Alexander et al., 2013; Fox and Alexander, 2015). These seasonal drivers influence significant biomass fluxes in the river flood plain system, resulting in large densities of wildlife populations congregating at the only permanent surface water resource in the dry season and dispersing in the wet season to access water in ephemeral pans (Alexander et al., 2012). Shared water resources, such as the dryland Chobe River, are under enormous pressure to sustain growing human and wildlife populations.

### Water quality and microbiological assessments

Water grab samples (1 L) were collected bimonthly from transect sites (*n* = 28 sampling points; Figure 1) located at 1 km intervals along the Chobe River between July 2011 and April 2012. Water quality assessments were conducted to determine *E. coli* (CFU/100 ml), fecal coliform (FC, CFU/100 ml) and TSS (mg/L) concentrations in the Chobe River following methodology previously described in Fox and Alexander (2015). Briefly, water grab samples were collected and processed within 24 h following United States Environmental Protection Agency (USEPA) methods 1103.1 and 1604 for *E. coli* enumeration (USEPA, 2000; Oshiro, 2002) and method 160.2 for TSS (USEPA, 1983). TSS samples were vacuum filtered (Millipore AP-40; Thermo Fisher) and dried at 103–105°C for an hour and weighed, and this drying cycle was repeated until a constant weight was obtained. To enumerate and isolate *E. coli*, water samples were vacuum filtered through sterile gridded nitrocellulose membrane filters (0.45 μm pore size; Thermo Fisher Scientific, Waltham, Massachusetts, USA), then aseptically transferred to RAPID' E.coli2 agar plates (BIORAD, Hercules, California, USA) and incubated at 37°C for 24 h prior to being enumerated. Up to six putative *E. coli* colonies were picked from each water sample and grown overnight in tryptic soy broth. DNA was extracted from all samples using the standard boiling method involving detergent/heat lysis followed by ethanol precipitation. DNA samples were screened by PCR to confirm presence of the *E. coli malB* locus, as previously described for water and wildlife samples (Pesapane et al., 2013) and originally described in Candrian et al. (1991). We note that these criteria will generate rare false positives arising from *Klebsiella* and *Enterobacter* species, which are ubiquitous in water environments and are detected in mammal feces (Leclerc et al., 2001). Members of both genera are positive for beta-galactosidase, thus test positive for lactose fermentation on MacConkey agar, like *E. coli*. As well, some isolates from each genus are positive for beta-glucoronidase (Maheux et al., 2015), allowing them to present like *E. coli* on differential colorimetric assays such as RAPID' E.coli2 agar. Both *Klebsiella* and *Enterobacter* encode the *malB* locus, though *Enterobacter* differs sufficiently at the DNA sequence level that stringent PCR conditions are unlikely to yield false positive results. A recent study of AR in surface water identified highly multi-drug resistant isolates of *Klebsiella* and *Enterobacter*, but at significantly lower numbers than *E. coli* (Zarfel et al., 2017), confirming that *Klebsiella* and *Enterobacter* can contribute to the environmental pool of AR genes, but their presence in surface water is generally unlinked to fecal contamination and these genera are unlikely to pose health risks to humans and other animals (Leclerc et al., 2001).

Positive isolates (*n* = 1997; Table 1) were tested against a panel of 10 antibiotics spanning six antibiotic classes: ampicillin (10 g; AMP10; penicillin), ceftiofur (30 g; XNL30; cephalosporin), chloramphenicol (30 g; C30; aminoglycoside), ciprofloxacin (5 g; CIP5; flouroquinolone), doxycycline (30 g; D30; tetracycline), gentamycin (10 g; G10; aminoglycoside), neomycin (30 g; N30; aminoglycoside), streptomycin (10 g; S10; aminoglycoside), sulfamethoxazole-trimethoprim (25 g; SXT25; sulfonamide), and tetracycline (30 g; T30; tetracycline) (BBL, Becton Dickinson Company) using the Kirby-Bauer disk diffusion method as described in Jobbins and Alexander (2015). These antibiotics were chosen because they are commonly used and are available in this region. Ceftiofur is routinely used in veterinary medicine providing insight into this aspect of AR development. The same panel of 10 antibiotics was used previously in this region to study microorganism transmission among humans, wildlife, and domestic animals (Pesapane et al., 2013; Jobbins and Alexander, 2015).

**Table 1:** Number of *Escherichia coli* isolates collected per river transect point and sampling date.

**Transect**																													
**Sampling periods**	**1**	**3**	**5**	**7**	**9**	**11**	**13**	**15**	**17**	**19**	**21**	**23**	**25**	**27**	**29**	**31**	**33**	**35**	**37**	**39**	**41**	**43**	**45**	**47**	**49**	**51**	**53**	**55**	**Grand total**
13-Jul-11	1	6	5	5	6	5	6	5	4	4	6	5	5	4	4	5	6	5	6	6	5	6	4	4	6	6	5	6	141
30-Jul-11	6	6	6	4	2	4	4	6	6	6	6	6	6	6	6	6	6	4	5	6	6	5	6	4	2	5	4	4	143
11-Aug-11	3	6	4	5	4	5	6	6	5	2	4	3	4	5	5	6	5	6	4	6	5	4	6	6	6	6	5	6	138
24-Aug-11	6	5	5	6	HP	2	5	6	5	6	5	6	5	5	4	6	4	6	6	5	4	3	6	6	5	C	6	C	128
8-Sep-11	4	6	5	6	HP	4	5	4	6	6	3	N	4	5	6	N	5	5	4	6	4	5	5	4	5	2	4	4	117
21-Sep-11	L	L	L	L	HP	L	L	L	L	L	L	6	3	5	3	4	6	4	6	5	1	N	4	4	2	2	3	4	62
6-Oct-11	2	3	6	6	HP	6	6	6	6	5	4	4	6	5	1	6	6	4	4	2	5	3	5	6	3	3	6	4	123
20-Oct-11	HP	6	3	4	HP	4	5	5	5	5	6	6	2	4	4	2	6	3	5	6	6	5	5	6	4	C	5	C	112
3-Nov-11	6	6	6	6	HP	6	6	5	4	6	6	6	5	5	5	6	6	6	6	6	6	4	6	6	1	6	6	6	149
17-Nov-11	5	6	6	5	HP	6	6	4	5	6	6	6	4	6	5	5	6	5	4	6	5	5	6	6	6	5	4	6	145
28-Nov-11	5	HP	5	6	HP	6	6	6	6	6	6	6	6	6	6	4	6	6	5	4	4	6	4	5	6	6	5	4	141
16-Dec-11	N	N	N	2	HP	6	6	4	5	2	4	5	5	L	L	L	L	L	L	L	1	L	6	L	L	L	L	L	46
4-Jan-12	B	B	B	B	B	B	B	B	B	B	B	B	B	B	B	6	6	6	5	6	6	5	5	6	5	6	5	6	73
18-Jan-12	6	6	5	6	HP	6	6	6	HP	HP	HP	6	6	5	3	6	6	6	6	6	4	3	5	5	N	6	6	6	126
14-Feb-12	5	3	2	3	HP	3	5	2	2	1	3	3	N	1	B	B	B	B	B	B	B	B	B	B	B	B	B	B	33
6-Mar-12	HP	3	5	3	HP	5	2	6	HP	3	HP	6	6	4	5	6	5	6	3	4	5	6	4	6	4	5	6	1	109
11-Apr-12	B	B	B	B	B	B	B	B	B	B	B	4	6	5	3	3	4	6	5	6	6	4	6	6	5	4	4	6	83
25-Apr-12	6	6	6	6	HP	6	5	5	5	6	6	B	B	B	B	5	6	6	6	6	6	6	6	6	6	3	5	4	128
Grand total	55	68	69	73	12	74	79	76	64	64	65	78	73	71	60	76	89	84	80	86	79	70	89	86	66	65	79	67	1997

*Transect 9 was habitually missed due to the presence of a resident hippopotamus (Hippopotamus amphibius) pod (HP). Other missing values were also due to HP presence, lack of access to a boat during that sample period (B), contamination of a bacterial sample (C), no E. coli being identified on confirmatory PCR (N), or a sample being misplaced (L)*.

Resistance data from water *E. coli* isolates were compared to wildlife and domestic animal data (June–September 2011, *n* = 440), and clinical human fecal data (*n* = 113), previously reported (Jobbins and Alexander, 2015) in our study area. Wildlife and domestic animal species include water-associated species [crocodile (*Crocodylus niloticus*), hippopotamus (*Hippopotamus amphibius*), African clawless otter (*Aonyx capensis*), and waterbuck (*Kobus ellipsiprymnus*)], as well as non-water-associated animals [African elephant (*Loxodonta africana*), banded mongoose (*Mungos mungo*), bushbuck (*Tragelaphus scriptus*), Cape buffalo (*Syncerus caffer*), Chacma baboon (*Papio ursinus*), domestic cattle (*Bos primigenius*), giraffe (*Giraffa camelopardalis*), greater kudu (*Tragelaphus strepsciseros*), guineafowl (*Numida meleagris*), impala (*Aepyceros melampus*), leopard (*Panthera pardus*), sable (*Hippotragus niger*), spotted hyena (*Crocuta crocuta*), vervet monkey (*Chlorocebus pygerythrus*), and warthog (*Phacochoerus africanus*) (Jobbins and Alexander, 2015)].

### Statistical analysis

Our study site is characterized by diverse land use types, which may influence AR dynamics. In this study, land type was therefore broken down and defined as “Park” for transects within the boundaries of the Chobe National Park, “Town” for transects occurring within the urban center (Kasane), or “Mixed Use” for transects in the growing, peri-urban area of Kazungula that incorporates land used for farming, wildlife corridors, and human settlement. All water-sampling observations were recorded between July 2011 and April 2012 at 15-day intervals. Based on rainfall during our study period, we classified the dry season as 13th July 2011 to 28th November 2011 and the wet season as 16th December 2011 to 25th April 2012. Statistical analyses were carried out using R 3.1.3 (R Core Team, 2014).

#### AR, water quality dynamics, and environmental predictors

To investigate the association between AR in *E. coli* samples and environmental predictors, including land use type and season in the sampling location, we used a combination of linear models. Ordinary least squares (OLS) linear regression was used to estimate the effect of environmental predictors on AR isolates in a given sample (Table 2). Separate OLS regression models were fit for wet and dry season data. Model residuals were assessed for linearity and normal error distribution using quantile-quantile plots, and Levene's test for equal variance, and a natural-log transformation was applied to mean AR and water quality perimeters (*E. coli* and TSS) to better approximate a normal distribution prior to fitting the regression models. FC was not included in the analysis because it strongly covaried with *E. coli*. We assessed for spatial autocorrelation (SAC) in model residuals using the Global Moran's I statistic with row-standardized weights, which was not significant for either the dry or wet season indicating SAC was not influential in the model residuals.

**Table 2:** Ordinary least squares regression of mean antibiotic resistance (AR) in waterborne *Escherichia coli* by sampling month and land use.

	**Dry season**				**Wet season**			
**Variable**	**Coefficient**	**Std. error**	***t-*value**	***p*-value**	**Coefficient**	**Std. error**	***t*-value**	***p*-value**
ln_E. coli	0.054	0.034	1.603	0.110	0.153	0.029	5.228	<0.001
ln_TSS	−0.100	0.045	−2.192	0.029	−0.098	0.040	−2.424	0.016
**Month**	**(reference is April)**
July	−0.155	0.083	−1.862	0.064	–	–	–	–
August	−0.265	0.085	−3.133	0.002	–	–	–	–
September	−0.143	0.100	−1.430	0.154	–	–	–	–
October	0.300	0.099	3.028	0.003	–	–	–	–
December	–	–	–	–	0.039	0.108	0.360	0.719
January	–	–	–	–	−0.067	0.068	−0.983	0.327
February	–	–	–	–	−0.110	0.111	−0.995	0.321
March	–	–	–	–	0.185	0.079	2.342	0.020
**Land Use**	**(reference is Town)**
Park	−0.113	0.072	−1.585	0.114	−0.280	0.072	−3.875	< 0.001
Mixed	−0.310	0.064	−4.830	< 0.001	−0.223	0.078	−2.849	0.005

*E. coli concentrations had a significant positive association with mean AR in the wet season but not in the dry season. Higher total suspended solids (TSS) was negatively associated with mean AR in both the wet and dry seasons. Mean AR was significantly higher in October and March, and significantly lower in August compared to levels seen at the beginning of the dry season (April). Mean AR was also significantly lower at transects in the Park and Mixed land use compared to Town transects*.

Additional linear regressions were run to determine whether similar trends held when considering the mean number of antibiotics to which an isolate was resistant (ranging from 0 to 10). A generalized linear model (GLM) was used to determine the effect of environmental predictors on the outcome of AR for each isolate. Once non-significant predictors were eliminated, a linear mixed model was used to account for the potential correlation between isolates within a given water sample through the assignment of random intercepts associated with each of the 414 water samples used in the regression.

Spatiotemporal patterns of mean AR and water quality perimeters (*E. coli*, FC and TSS) were interpolated and mapped using empirical Bayesian kriging (EBK) in ESRI ArcGIS v.10.2 (Environmental Systems Research Institute, Redlands, California). EBK makes predictions through automated subsetting and simulation using a restricted maximum likelihood approach to obtain an optimal empirical semivariogram and calculate weights such that the interpolation mean square error is minimized (Pilz and Spöck, 2008). Each iteration of EBK uses semivariogram models defined by nearby values, rather than being influenced by more distant points, allowing for more accurate predictions even when the data are moderately non-stationary (Krivoruchko, 2012). A standard search radius of 10,000 m was used to ensure a minimum of 10 and a maximum of 15 neighbors were included in the interpolation. Model suitability was assessed based upon the criteria that root-mean-square error (RMSE) was minimized and the root-mean square standardized error was close to one (Table 3). The resulting maps show predicted mean AR (Figure 3), and the concentrations of *E. coli*, FC and TSS (Figure 4) in water samples collected from a given location. A heat map of AR, *E. coli* and TSS isolated from water samples is shown by sampling date and transect (Figure 5) to illustrate location and timing of water quality declines and AR emergence.

**Table 3:** Cross validation results for wet and dry season semivariogram models used in empirical Bayesian kriging (EBK) predictions.

**Variable**	**Semivariogram type**	**RMSE**	**RMSE standardized**
Wet season meanAR	Power	0.370	0.967
Dry season meanAR	Power	0.337	0.981
Wet season *E. coli*	Power	27.2	1.01
Dry season *E. coli*	Power	10.3	0.904
Wet season FC	Power	41.8	0.934
Dry season FC	Power	23.6	0.945
Wet season TSS	Power	3.79	0.864
Dry season TSS	Power	1.07	0.739

*A root mean square error (RMSE) standardized close to one indicate improved model performance, with highest predictive accuracy seen for dry season mean antibiotic resistance (AR) and the poorest performance observed for dry season total suspended solids (TSS)*.

#### AR profiles across sample type and land use

In order to determine relationships between AR in isolates from surface water and AR isolates found in humans and wildlife sharing the same water source, we conducted a Spearman rank correlation in R. Spearman rank correlation coefficients were calculated by comparing AR in water samples (*n* = 1997) to AR in *E. coli* isolates from fecal samples collected from humans (*n* = 113), non-water-associated animals (*n* = 382), and water-associated animals [*n* = 58, previously published data, see (Jobbins and Alexander, 2015)]. The relationships between AR profiles by sample type and land use are visualized in Figure 6.

## Results

### Effects of land use and season on surface water AR dynamics

To explore AR dynamics, we examined the distribution and frequency of resistance phenotypes in *E. coli* in our sample area over a 10-month period, spanning wet and dry seasons. By land use, the mean level of resistance (number of antibiotics to which an isolate demonstrated resistance) was 1.39 (*SD* = 0.051) in the Park, 2.15 (*SD* = 0.098) in Town, and 1.76 (*SD* = 0.079) in the Mixed land use areas.

**Figure 2:**
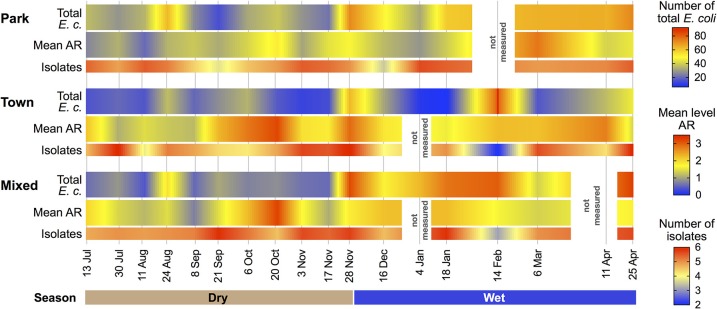
Mean water *Escherichia coli* isolates exhibiting antibiotic resistance (AR), corresponding total *E. coli* counts and number of *E. coli* isolates obtained during water quality assessments within each land use type and sampling period.

When examining differences in mean AR, ordinary linear models identified both land use and season as being statistically significant predictors, with the wet season resulting in higher mean AR than the dry season (coeff = 0.215; *t* = 2.938; *p* = 0.003). Isolates from the Park exhibited lower levels of AR than Mixed Use (coeff = −0.430; *t* = −5.184; *p* < 0.001) and Town, which exhibited the highest levels of AR (coeff = 0.322; *t* = 3.130; *p* = 0.002; Figure 2). A linear mixed model was then used to evaluate these relationships accounting for the expected correlation among isolates (*n* = 1997) distributed among water samples (*n* = 414). Similar to the results of the OLS, Analysis of Variance (ANOVA) of the most parsimonious model revealed significance of both land use type (*F* = 18.633) and season (*F* = 4.144) in the mean number of antibiotics to which an isolate exhibited resistance.

### Spatial patterns of AR, *E. coli*, and TSS

**Figure 3:**
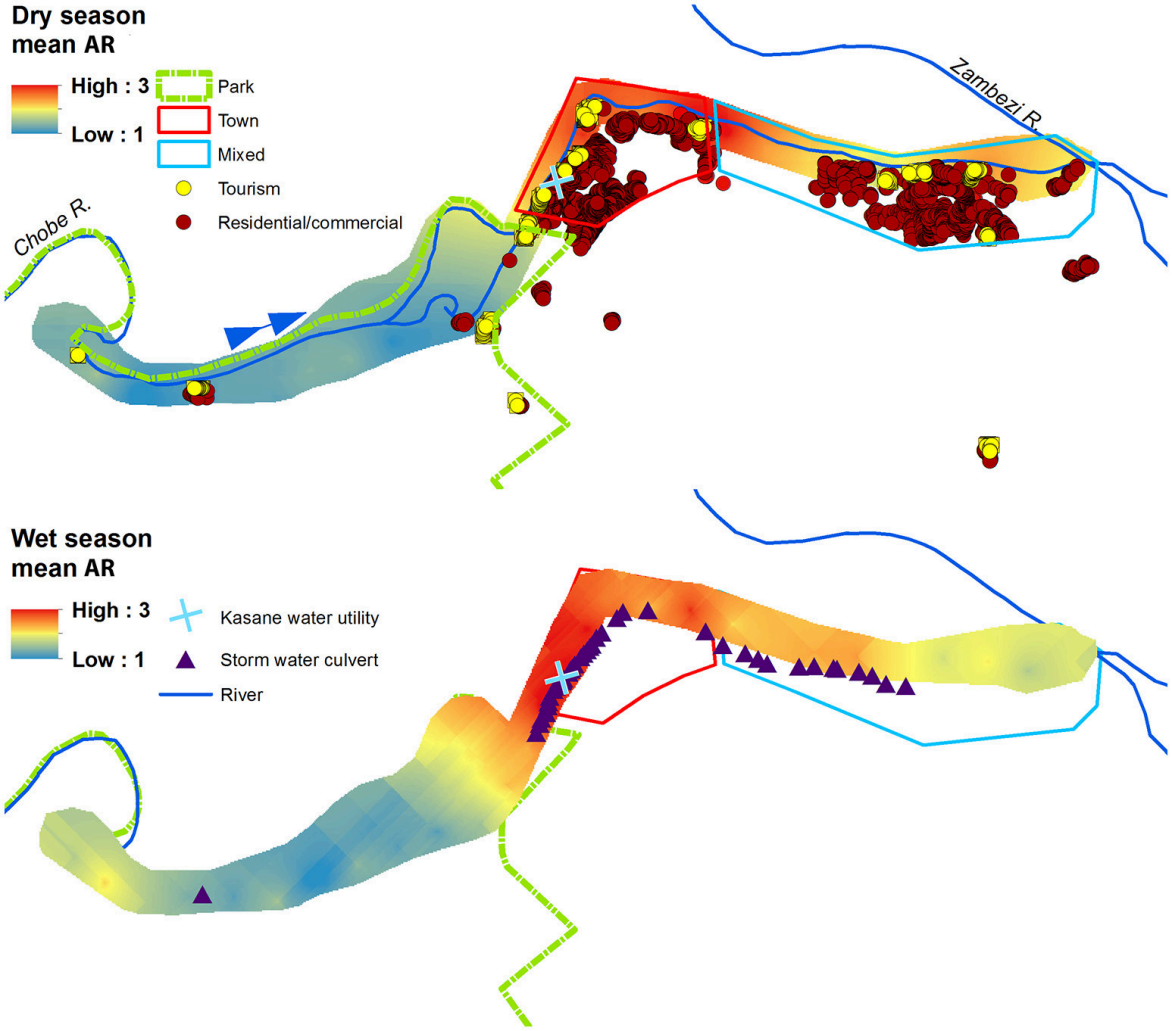
Prediction surfaces of mean wet and dry season antibiotic resistance (AR) interpolated using empirical Bayesian kriging (EBK). Park (green border), Town (red border), and Mixed (light blue border) land use boundaries are shown, along with commercial, residential, and tourism buildings, and storm water culverts. The blue arrow indicates the direction of Chobe River flow.

Using empirical Bayesian kriging, we evaluated the spatiotemporal patterns of AR, *E. coli*, FC and TSS (Figure 3). A summary of root mean square error (RMSE) associated with semiovariogram models estimated using empirical Bayesian kriging (EBK) are shown in Table 3. The RMSE are the cross validation results for wet and dry season semivariogram models used in EBK predictions. Lower model RMSE and a RMSE standardized close to one indicate improved model performance. A RMSE standardized greater than one indicates underestimation of the variability in our predictions, while < 1 indicates overestimation of the variability.

**Figure 4:**
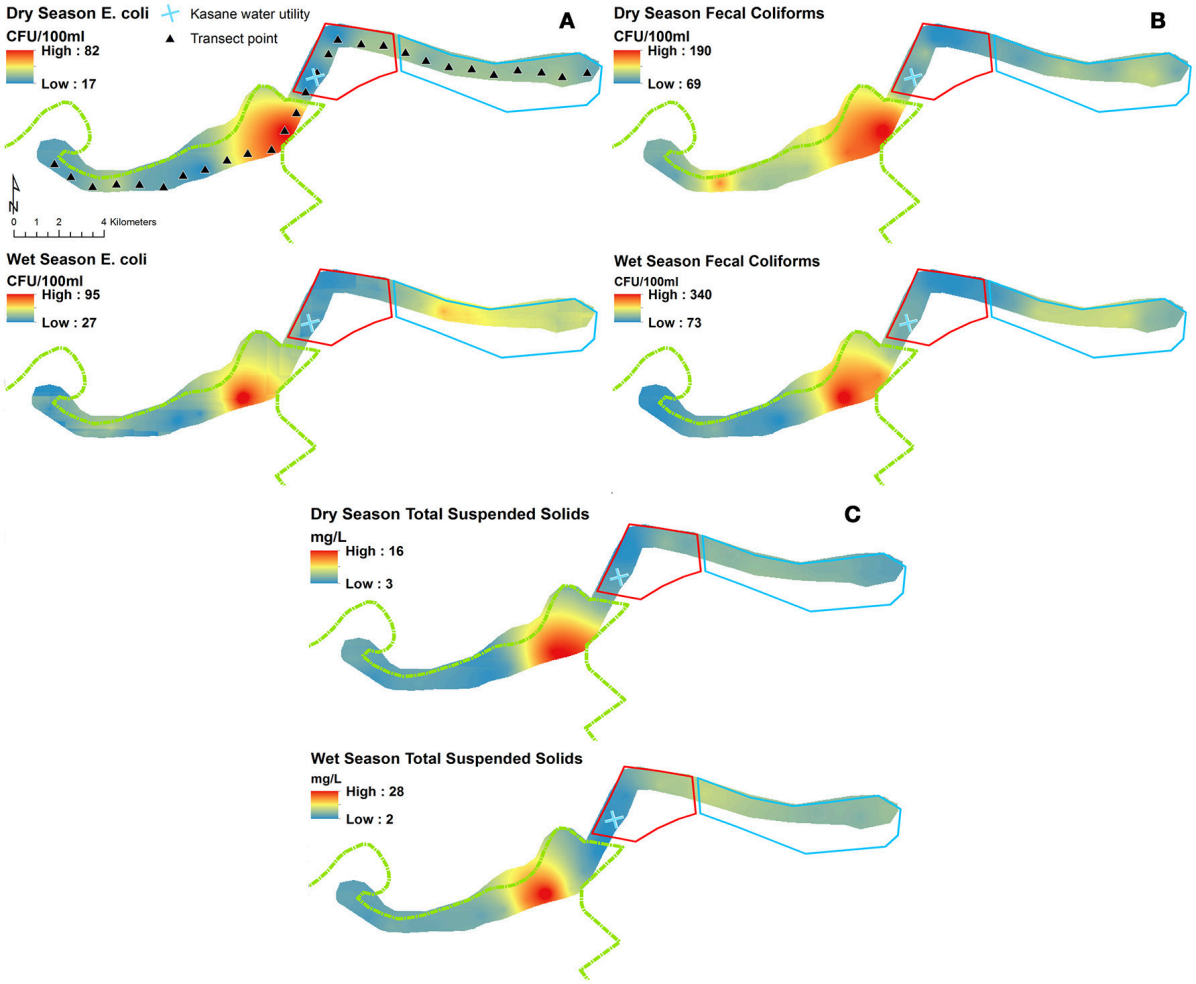
Prediction surfaces of wet and dry season **(A)** *Escherichia coli* (CFU/100 ml), **(B)** fecal coliforms (FC; CFU/100 ml), and **(C)** total suspended solids (TSS; mg/L) interpolated using empirical Bayesian kriging. Park (green border), Town (red border), and Mixed (light blue border) land use boundaries are shown.

**Figure 5:**
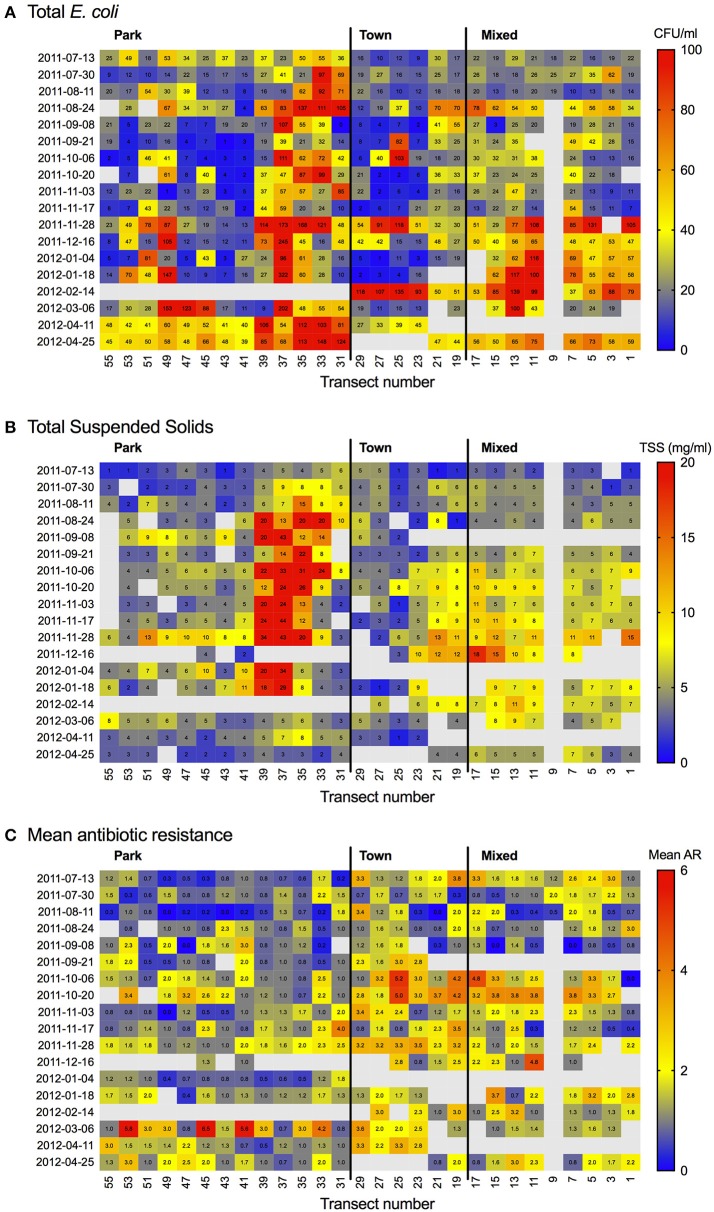
Heat map showing **(A)** *Escherichia coli* (CFU/100 ml), **(B)** total suspended solids (TSS; mg/L) and **(C)** Mean antibiotic resistance (AR) by transect and sampling date.

The spatial and temporal trends of AR, *E. coli*, FC and TSS were plotted to visualize how concentrations change along the river (Figures 3, 4 (spatial) and Figure 5 (temporal and spatial)). Maps for both wet and dry season mean AR show how bacterial isolates in water grab samples collected from the river in the town of Kasane had the highest resistance, with significantly lower mean AR observed in the Park and Mixed land use (Figure 3). In the wet season, the region of high AR extended further upstream into the Park compared to in the dry season. Numerous storm water drains are also located in the region of elevated AR, but it is unclear how these structures contribute to AR distribution in surface water. Evaluating the spatiotemporal patterns in *E. coli*, FC and TSS from water quality grab samples showed that the area of the river in the Chobe National Park just upstream of Town transects had the highest concentrations during both the wet and the dry seasons (Figure 4). During the wet season, water samples from transects in Mixed land use also had higher predicted concentrations of *E. coli*, FC, and TSS.

### Comparison of AR profiles in water, animals and humans across land use

**Figure 6:**
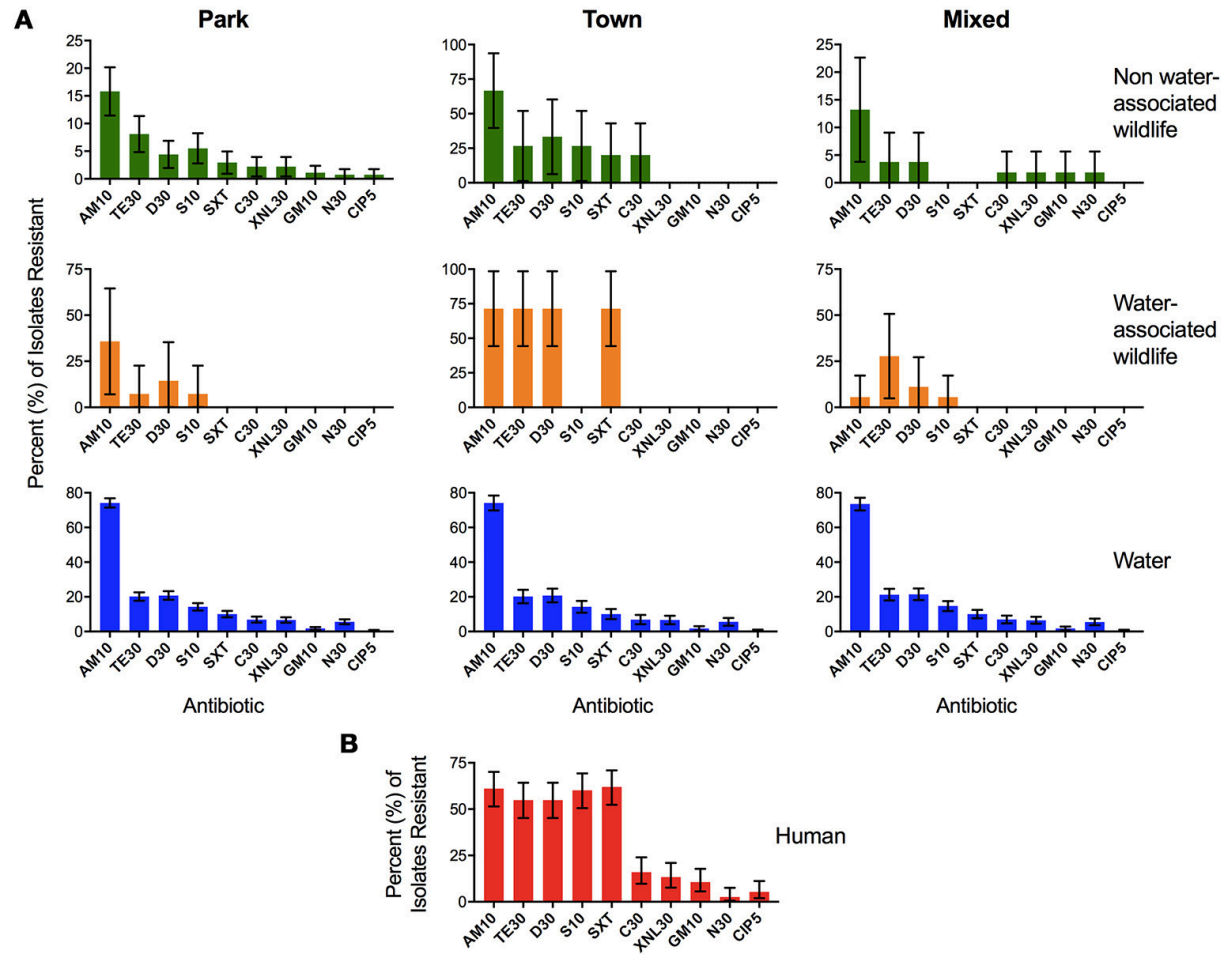
Percent (%) of *Escherichia coli* isolates resistant to each antibiotic in the four different sample types: **(A)** water, water-associated animals, non-water-associated animals, and **(B)** human fecal samples. The panel of antibiotics used to test the isolates included ampicillin (AM10), chloramphenicol (C30), ciprofloxacin (CIP5), doxycycline (D30), gentamicin (GM10), neomycin (N30), streptomycin (S10), tetracycline (TE30), trimethoprim-sulfamethoxazole (SXT25), and Ceftiofur (XNL30). Samples from water–associated animals included crocodile (*Crocodylus niloticus*), hippopotamus (*Hippopotamus amphibius*), African clawless otter (*Aonyx capensis*), and waterbuck (*Kobus ellipsiprymnus*) (Jobbins and Alexander, 2015). Samples from non-water-associated animals included African elephant (*Loxodonta africana*), banded mongoose (*Mungos mungo*), bushbuck (*Tragelaphus scriptus*), Cape buffalo (*Syncerus caffer*), Chacma baboon (*Papio ursinus*), giraffe (*Giraffa camelopardalis*), greater kudu (*Tragelaphus strepsciseros*), guineafowl (*Numida meleagris*), impala (*Aepyceros melampus*), leopard (*Panthera pardus pardus*), sable (*Hippotragus niger*), spotted hyena (*Crocuta crocuta*), vervet monkey (*Chlorocebus pygerythrus*), and warthog (*Phacochoerus africanus*) (Jobbins and Alexander, 2015). Bars represent the 95% binomial proportion confidence intervals.

Mean AR in isolates from human fecal samples (*n* = 113) was compared to mean AR in *E. coli* isolates from fecal samples collected from non-water-associated animals (*n* = 382), water-associated animals [*n* = 58, previously published data, see (Jobbins and Alexander, 2015)], and water samples (*n* = 1997). A similar rank order of resistance to antibiotics was observed across sample types and land use (Figure 6), with high Spearman rank correlation coefficients between resistance in water across land uses (*R* = 1, *p* < 0.0001) indicating that the river is well-mixed and relatively homogeneous along this stretch, and between human isolates and water isolates (*R* = 0.8, *p* < 0.001). Antibiotic resistance in water-associated animals demonstrated the strongest positive association with water AR profiles in the Park (*R* = 0.88, *p* < 0.0001), followed by Mixed (*R* = 0.82, *p* < 0.001) then Town (*R* = 0.7, *p* < 0.05). Non-water-associated wildlife AR values in the Town and Park land areas were highly correlated with that observed in water (*R* = 0.96 and 0.94, *p* < 0.001), while non-water-associated wildlife in the Mixed land type was not associated with water AR profiles (*R* = 0.57, *p* < 0.05).

## Discussion

In this study, we identify widespread movement of AR in surface water across land use in the absence of commercial livestock production systems and large medical facilities. This suggests that direct human inputs together with other environmental factors may be sufficient for the extensive dispersal of AR observed in this ecosystem across protected and unprotected landscapes. Season and land use were both important predictors of mean AR in surface water with greater AR levels observed in the wet season than in the dry season (*p* = 0.003). Our results suggest that even in the absence of agricultural inputs, human source AR movement may be sufficient to create widespread AR dispersal across surface water even in protected areas upstream of urban centers.

### Land use and AR

#### Urban and peri-urban landscapes

The urban landscape (Town) had the highest level of AR *E. coli* across land types (Figure 2, μ = 2.145, *SD* = 0.098). The area with the highest levels of AR was located along a segment of the river in Town associated with a peak density of low-income residential properties (264 residences/ha). In this area, an estimated 69% of households use traditional, self-built pit latrines, instead of flush toilets (Alexander and Godrej, 2015). Pit latrines have been previously linked to water quality declines through contamination of groundwater (Jacks et al., 1999; Ahaneku and Adeoye, 2014). Our work suggests that these impacts may be even greater in areas where health-seeking behaviors lead to the use of pit latrines as wastewater receptacles to avoid contaminating residential living areas. Disposal of greywater into the pit latrine, particularly where the ratio of households to pit latrine is high, resulting in hydraulic overloading of the pit latrine may lead to the leaching of human fecal waste and AR bacteria (commensal and pathogenic) into the surrounding soil and groundwater with subsurface movement into nearby surface water resources (Alexander and Godrej, 2015). In the wet season, rising water tables may inundate previously unsaturated soils and further flush AR bacteria from environmental reservoirs, resulting in the elevated levels of AR *E. coli* seen in the Chobe River during this time period. Spatial patterns of AR concentration also appeared to be associated with storm water drain locations suggesting that the flushing of these drains during heavy rainfall may also contribute to water quality declines observed during the wet season (Hsu et al., 2015) (Figures 3, 4).

Once in the environment, these bacteria can exchange ARGs and aid in the emergence of new AR bacteria in the resistome. This has been demonstrated *in vitro* with ARG transfer occurring between environmental *E. coli* isolates, *Citrobacter freundii*, and *Klebsiella pneumonia* (Mckeon et al., 1995). The use of pit latrines is reminiscent of pit systems and lagoons in animal agriculture, whereby fecal indicator bacteria, AR bacteria, and ARGs leach or flow into groundwater and river systems (Chee-Sanford et al., 2001; Sapkota et al., 2007; Li et al., 2014). In these situations, AR bacteria have been discovered in high concentrations in rural groundwater (60% of coliforms tested), with up to 33% of isolates resistant to five or more antibiotics (Mckeon et al., 1995). Movement through ground water can be significant with, for example, ARGs from a swine production facility using pit systems moving in ground water more than 250 m from the point of origin (Chee-Sanford et al., 2001).

The Mixed Use landscape had lower AR *E. coli* levels than in the urban land use, despite being located downstream. These differences may be due to a number of factors. Firstly, the number of low-income residential properties is significantly less (100 residences/ha) than in Town (Alexander and Godrej, 2015), and these residences are located on elevated land, farther away from the river. The groundwater table is much lower than in Town relative to the region of more concentrated residential areas. Secondly, the use of pit latrines to discard graywater is less prevalent in this region than in Town (Alexander and Godrej, 2015), potentially reducing the leaching of AR bacteria. The presence of the wastewater treatment plant in the Mixed Use land area may also contribute to the overall levels of AR, *E. coli*, and FC in the Chobe River in this region through leaching and run-off. Although levels were lower than in the urban land use, the concentrations of *E. coli*, FC and TSS in the mixed land use increased significantly during the wet season.

#### AR in protected areas upstream of human populations

Direct human influences do not explain the levels of AR seen in the Park, which is located upstream from known sources of human fecal waste. The Park land use is characterized by extensive floodplains where high *E. coli*, FC and TSS were identified (Figure 4). We previously found a strong association between floodplain habitats and high *E. coli* levels in the river, with water quality declines significantly associated with wildlife fecal counts (Fox and Alexander, 2015). Significant levels of AR in the wildlife population has previously been identified (Pesapane et al., 2013; Jobbins and Alexander, 2015) with AR levels in *E. coli* significantly higher in synanthropic wildlife (i.e., banded mongoose) carnivores (i.e., leopard), and water-associated species (i.e., hippopotamus and otter) (Jobbins and Alexander, 2015). These data suggest life history strategy may be key to understanding exposure and transmission dynamics (Jobbins and Alexander, 2015).

The flood pulse may also play a central role in AR dynamics. Slow-moving floodwaters travel thousands of kilometers along the Zambezi and Cuando/Chobe Rivers, arriving at the start of the dry season and eventually backing up behind the Mambova fault line, which lies several meters higher than the surrounding floodplain. In some years, floodwaters can inundate a massive area covering several thousand square kilometers, maintaining high water levels well into the dry season, when all other natural surface water sources have evaporated. Flooding may result in the mobilization of AR bacteria out of existing biofilms in floodplain sediments and groundwater reservoirs into the water column, resulting in the significant spike in AR *E. coli* levels during the wet season. At freshwater beaches, fecal coliforms (enterococci) and *E. coli* abundances in the top 20 cm of wet sand have been known to exceed the amount in the water column by a factor of 38 (Wheeler Alm et al., 2003). Furthermore, AR elements have been discovered in floodplain sediment down to a depth greater than 80 cm, indicating that these elements may persist for 30–40 years (Tamtam et al., 2011). The spike in waterborne AR *E. coli* levels observed in October coincides with minimum annual water levels in the Chobe River system and may be related to concentration of AR bacteria as water levels drop. Another potential contributing factor to increased AR levels observed at the end of the dry season may be the significant increase in wildlife densities that occur as the dry season advances and water resources dry up in the interior. Wildlife access to water is limited due to developments along the riverfront, concentrating wildlife use into smaller areas focused in the National Park with consequent increases in fecal inputs and water quality declines in the segment upriver of the water intake for the population (Fox and Alexander, 2015).

### Water quality and AR dynamics

Water quality variables and surface water AR are significantly influenced by season and land use. In our study system, water quality declines are known to be influenced by wildlife distribution in the dry season, and rainfall and river hydrology in the wet season (Fox and Alexander, 2015). Here, in-river *E. coli* concentrations had a significant positive association with mean AR in the wet season but not in the dry season (Table 2). Increases in *E. coli* are known to be associated with rainfall and river height (Alexander et al., 2018), with overland movement and runoff of fecal waste likely contributing to water quality declines (increases in *E. coli* and FC) and AR levels in the wet season. Across all land uses and both seasons, Town exhibited the best water quality perimeters overall (lowest *E. coli*, FC and TSS concentrations; Figure 4), yet the highest mean AR levels (Figures 2, 3). TSS was negatively associated with mean AR in both the wet and dry seasons (Table 2). To our knowledge this is the first study integrating water quality studies and AR across landscapes. Our data suggests important complexity exists signaling the significance of human- environmental couplings on AR movement and accumulation.

### Human use of the river and AR circulation

Classified as a “biodiversity hotspot,” the abundance of wildlife in the Chobe region attracts more than 20,000 tourists annually. The concentrated recreational use of the Chobe River in the Chobe National Park and high density of boats may contribute to the spread of AR into this protected area. This boat activity, combined with low water levels in the dry season, may result in the increased re-suspension of river sediment reservoirs of AR *E. coli*. In addition, human effluent from boats entering the park may enter the system if the effluent is not disposed of properly or if existing holding tanks over flow, potentially providing a mechanism for additional AR inputs into the system. Numerous studies have shown that tourists can aid in the contraction and distribution of pathogens and AR microbes worldwide (reviewed in Okeke and Edelman, 2001). Indeed, AR *E. coli* isolates were found in fecal samples from tourists visiting Mexico without ever having consumed prophylactic antibiotics prior to traveling (Murray et al., 1990).

### Antibiotic profiles across the human, wildlife, and environmental interface

AR profiles of *E. coli* from surface water, humans and wildlife populations from the Chobe region were highly similar (Figure 5). These data indicate the need for caution in ascribing AR threats as arising dominantly from agricultural inputs (House, 2015) and highlights the need to better understand the manner in which direct human-environmental couplings may contribute to resistance dissemination across the environment. Previous work utilizing whole-genome sequencing has revealed that there is extensive sharing of AR genes between human, animal and environmental microbial communities (Pehrsson et al., 2016). This sharing between bacteria and microbial communities is assisted by horizontal gene transfer via mobile genetic elements and AR clusters (Pehrsson et al., 2016), increasing the likelihood that these elements may reach pathogenic organisms.

The ingestion of surface water harboring AR microbes has been identified as a likely route of AR movement in naïve wildlife populations (Mariano et al., 2009; Jobbins and Alexander, 2015). In South Africa, impala drinking from rivers containing AR were shown to be 19 times more likely to have AR microbes than impala drinking from uncontaminated rivers (Mariano et al., 2009). However, in our study site, impala, and other water-dependent herbivores (buffalo, kudu, etc.) showed little to no AR, signifying that ingesting water alone was insufficient for transmission of resistance. Instead, water-associated wildlife (hippopotamus, otters, etc.) had higher levels of AR resistance, indicating that the duration spent in the water, and/or consumption of water-dwelling vegetation or associated sediments may also play a role (Jobbins and Alexander, 2015).

The dissemination of AR in the environment is a key component of the growing global health threat, however, surveillance of environmental antimicrobial hot spots is absent from action plans. Perennial surface water affords ecosystem level surveillance, allowing early detection and control of AR before spreading into clinically important pathogens. Indeed, water quality declines represent the penultimate expression of system degradation. It is clear from our study that surveillance focused on ARG in human and animal pathogens may have limited effectiveness. This surveillance approach simply alerts us to the ultimate failure in controlling AR among pathogens that threaten human health.

## Conclusion

Our study provides clear indication that humans have the potential to directly influence the movement and accumulation of AR across protected and unprotected landscapes in the absence of commercial livestock production and extensive health facilities. Focused and expensive efforts to minimize pollution from agricultural sources, while important, may only have incremental benefits to the management of antimicrobial resistance across complex landscapes. AR control efforts might benefit from sentinel surveillance systems that identify ARG movement and accumulation in the environment before invasion into pathogenic microbial communities. Controlling direct human AR input into the environment remains a critical challenge.

## Author contributions

CS conducted the laboratory work, interpreted the data, and drafted and revised the manuscript. ED conducted the statistical analyses, aided in data interpretation and wrote the method and results sections corresponding to the statistical analyses. JF conducted the spatial analysis and wrote the corresponding sections in the manuscript. AC interpreted the data and assisted in manuscript revision. KA developed the study concept and design, interpreted the data and conducted critical revision of the manuscript, and secured funding for the study.

### Conflict of interest statement

The authors declare that the research was conducted in the absence of any commercial or financial relationships that could be construed as a potential conflict of interest.

## References

[B1] Ahaneku I. E. Adeoye P. A. (2014). Impact of pit latrines on groundwater quality of fokoslum, Ibadan, Southwestern Nigeria. Br. J. Appl. Sci. Technol. 4, 440–449. 10.9734/BJAST/2014/5079

[B2] Alexander K. A. Blackburn J. K. (2013). Overcoming barriers in evaluating outbreaks of diarrheal disease in resource poor settings: assessment of recurrent outbreaks in Chobe District, Botswana. BMC Public Health 13:775. 10.1186/1471-2458-13-775 23971427 PMC3765974

[B3] Alexander K. A. Blackburn J. K. Vandewalle M. E. Pesapane R. Baipoledi E. K. Elzer P. H. (2012). Buffalo, bush meat, and the zoonotic threat of brucellosis in Botswana. PLoS ONE 7:e32842. 10.1371/journal.pone.0032842 22412932 PMC3297602

[B4] Alexander K. A. Carzolio M. Goodin D. Vance E. (2013). Climate change is likely to worsen the public health threat of diarrheal disease in Botswana. Int. J. Environ. Res. Public Health 10, 1202–1230. 10.3390/ijerph10041202 23531489 PMC3709313

[B5] Alexander K. A. Godrej A. (2015). Greywater disposal practices in Northern Botswana—the silent spring? Int. J. Environ. Res. Public Health 12, 14529–14540. 10.3390/ijerph121114529 26580640 PMC4661665

[B6] Alexander K. A. Heaney A. Shaman J. (2018). Flood pulse dynamics and surface water quality declines predict diarrheal disease identifying increased population vulnerability to climate change. PLoS Med. (Accepted). 10.1371/journal.pmed.1002688 PMC6224043 30408029

[B7] Allen H. K. Donato J. Wang H. H. Cloud-Hansen K. A. Davies J. Handelsman J. (2010). Call of the wild: antibiotic resistance genes in natural environments. Nat. Rev. Microbiol. 8, 251–259. 10.1038/nrmicro2312 20190823

[B8] Bartoloni A. Pallecchi L. Rodríguez H. Fernandez C. Mantella A. Bartalesi F.. (2009). Antibiotic resistance in a very remote Amazonas community. Int. J. Antimicrob. Agents 33, 125–129. 10.1016/j.ijantimicag.2008.07.029 18947984

[B9] Bouki C. Venieri D. Diamadopoulos E. (2013). Detection and fate of antibiotic resistant bacteria in wastewater treatment plants: a review. Ecotoxicol. Environ. Saf. 91, 1–9. 10.1016/j.ecoenv.2013.01.016 23414720

[B10] Candrian U. Furrer B. Höfelein C. Meyer R. Jermini M. Lüthy J. (1991). Detection of Escherichia coli and identification of enterotoxigenic strains by primer-directed enzymatic amplification of specific DNA sequences. Int. J. Food Microbiol. 12, 339–351. 10.1016/0168-1605(91)90148-I 1854602

[B11] Chee-Sanford J. C. Aminov R. I. Krapac I. Garrigues-Jeanjean N. Mackie R. I. (2001). Occurrence and diversity of tetracycline resistance genes in lagoons and groundwater underlying two swine production facilities. Appl. Environ. Microbiol. 67, 1494–1502. 10.1128/AEM.67.4.1494-1502.2001 11282596 PMC92760

[B12] Czekalski N. Gascón Díez E. Bürgmann H. (2014). Wastewater as a point source of antibiotic-resistance genes in the sediment of a freshwater lake. ISME J. 8, 1381–1390. 10.1038/ismej.2014.8 24599073 PMC4069405

[B13] Davies J. (2007). Microbes have the last word. EMBO Rep. 8, 616–621. 10.1038/sj.embor.7401022 17603533 PMC1905906

[B14] Fox J. T. Alexander K. A. (2015). Spatiotemporal variation and the role of wildlife in seasonal water quality declines in the Chobe River, Botswana. PLoS ONE 10:e0139936. 10.1371/journal.pone.0139936 26460613 PMC4603952

[B15] House T. W. (2015). National Action Plan for Combating Antibiotic-Resistant Bacteria. Washington, DC: The White House.

[B16] Hsu C. Y. Hsu B. M. Ji W. T. Chang T. Y. Kao P. M. Tseng S. F. (2015). A potential association between antibiotic abuse and existence of related resistance genes in different aquatic environments. Water Air Soil Pollut. 226, 1–9. 10.1007/s11270-014-2235-z

[B17] Jacks G. Sefe F. Carling M. Hammar M. Letsamao P. (1999). Tentative nitrogen budget for pit latrines Äìeastern Botswana. Environ. Geol. 38, 199–203.

[B18] Jobbins S. E. Alexander K. A. (2015). From whence they came - antibiotic-resistant *Escherichia coli* in African wildlife. J. Wildl. Dis. 51, 811–820. 10.7589/2014-11-257 26221860

[B19] Knapp C. W. Lima L. Olivares-Rieumont S. Bowen E. Werner D. Graham D. W. (2012). Seasonal variations in antibiotic resistance gene transport in the Almendares River, Havana, Cuba. Front. Microbiol. 3:396. 10.3389/fmicb.2012.00396 23189074 PMC3505016

[B20] Krivoruchko K. (2012). Empirical Bayesian Kriging. ESRI: Redlands. California: USA Available online at: http://www.esri.com/news/arcuser/1012/empirical-byesian-kriging.html (Accessed February 08, 2016).

[B21] Leclerc H. Mossel D. Edberg S. Struijk C. (2001). Advances in the bacteriology of the coliform group: their suitability as markers of microbial water safety. Annu. Rev. Microbiol. 55, 201–234. 10.1146/annurev.micro.55.1.201 11544354

[B22] Li X. Watanabe N. Xiao C. Harter T. Mccowan B. Liu Y.. (2014). Antibiotic-resistant *E. coli* in surface water and groundwater in dairy operations in Northern California. Environ. Monitor. Assess. 186, 1253–1260. 10.1007/s10661-013-3454-2 24097011

[B23] Lima-Bittencourt C. I. Cursino L. Gonçalves-Dornelas H. Pontes D. S. Nardi R. M. Callisto M.. (2007). Multiple antimicrobial resistance in Enterobacteriaceae isolates from pristine freshwater. Genet. Mol. Res. 6, 510–521. 17985304

[B24] Maheux A. F. Dion-Dupont V. Bouchard S. Bisson M. A. Bergeron M. G. Rodriguez M. J. (2015). Comparison of four β-glucuronidase and β-galactosidase-based commercial culture methods used to detect *Escherichia coli* and total coliforms in water. J. Water Health 13, 340–352. 10.2166/wh.2014.175 26042967

[B25] Mariano V. Mccrindle C. Cenci-Goga B. Picard J. (2009). Case-control study to determine whether river water can spread tetracycline resistance to unexposed Impala (*Aepyceros melampus*) in Kruger National Park (South Africa). Appl. Environ. Microbiol. 75, 113–118. 10.1128/AEM.01808-08 18978077 PMC2612227

[B26] Mckeon D. M. Calabrese J. P. Bissonnette G. K. (1995). Antibiotic resistant gram-negative bacteria in rural groundwater supplies. Water Res. 29, 1902–1908. 10.1016/0043-1354(95)00013-B

[B27] Murray B. Mathewson J. Dupont H. Ericsson C. Reves R. (1990). Emergence of resistant fecal *Escherichia coli* in travelers not taking prophylactic antimicrobial agents. Antimicrob. Agents Chemother. 34, 515–518. 10.1128/AAC.34.4.515 2188583 PMC171635

[B28] Okeke I. N. Edelman R. (2001). Dissemination of antibiotic-resistant bacteria across geographic borders. Clin. Infect. Dis. 33, 364–369. 10.1086/321877 11438903

[B29] Oshiro R. (2002). Method 1604: Total Coliforms and Escherichia coli in Water by Membrane Filtration Using a Simultaneous Detection Technique (MI Medium). Washington, DC: US Environmental Protection Agency.

[B30] Pehrsson E. C. Tsukayama P. Patel S. Mejía-Bautista M. Sosa-Soto G. Navarrete K. M.. (2016). Interconnected microbiomes and resistomes in low-income human habitats. Nature 533, 212–216. 10.1038/nature17672 27172044 PMC4869995

[B31] Pesapane R. Ponder M. Alexander K. (2013). Tracking pathogen transmission at the human–wildlife interface: banded mongoose and *Escherichia coli*. Eco Health 10, 115–128. 10.1007/s10393-013-0838-2 23612855

[B32] Pilz J. Spöck G. (2008). Why do we need and how should we implement Bayesian kriging methods. Stochastic Environ. Res. Risk Assess. 22, 621–632. 10.1007/s00477-007-0165-7

[B33] Pruden A. Arabi M. Storteboom H. N. (2012). Correlation between upstream human activities and riverine antibiotic resistance genes. Environ. Sci. Technol. 46, 11541–11549. 10.1021/es302657r 23035771

[B34] R Core Team (2014). R: A Language and Environment for Statistical Computing. Vienna: R Foundation for Statistical Computing Available online at: http://www.R-project.org/

[B35] Rizzo L. Manaia C. Merlin C. Schwartz T. Dagot C. Ploy M.. (2013). Urban wastewater treatment plants as hotspots for antibiotic resistant bacteria and genes spread into the environment: a review. Sci. Total Environ. 447, 345–360. 10.1016/j.scitotenv.2013.01.032 23396083

[B36] Sapkota A. R. Curriero F. C. Gibson K. E. Schwab K. J. (2007). Antibiotic-resistant enterococci and fecal indicators in surface water and groundwater impacted by a concentrated swine feeding operation. Environ. Health Perspect. 1040–1045. 10.1289/ehp.9770 17637920 PMC1913567

[B37] Schwartz T. Kohnen W. Jansen B. Obst U. (2003). Detection of antibiotic-resistant bacteria and their resistance genes in wastewater, surface water, and drinking water biofilms. FEMS Microbiol. Ecol. 43, 325–335. 10.1111/j.1574-6941.2003.tb01073.x 19719664

[B38] Tamtam F. Le Bot B. Dinh T. Mompelat S. Eurin J. Chevreuil M. (2011). A 50-year record of quinolone and sulphonamide antimicrobial agents in Seine River sediments. J. Soils Sediments 11, 852–859. 10.1007/s11368-011-0364-1

[B39] USEPA (1983). Methods for Chemical Analysis of Water and Wastes, EPA-600/4-79-020. Methods 160.2. Washington, DC: USEPA.

[B40] USEPA (2000). Improved Enumeration Methods for the Recreational Water Quality Indicators: Enterococci and Escherichia coli EPA-821/R97/004. Section 10.3 Modified E coli Method. Washington, DC: USEPA.

[B41] Van Den Bogaard A. E. Stobberingh E. E. (2000). Epidemiology of resistance to antibiotics: links between animals and humans. Int. J. Antimicrob. Agents 14, 327–335. 10.1016/S0924-8579(00)00145-X 10794955

[B42] Wheeler Alm E. Burke J. Spain A. (2003). Fecal indicator bacteria are abundant in wet sand at freshwater beaches. Water Res. 37, 3978–3982. 10.1016/S0043-1354(03)00301-4 12909116

[B43] World Health Organization (2001). WHO Global Strategy for Containment of Antimicrobial Resistance. World Health Organization.

[B44] World Health Organization (2014). Antimicrobial Resistance: 2014 Global Report on Surveillance. World Health Organization.

[B45] Zarfel G. Lipp M. Gürtl E. Folli B. Baumert R. Kittinger C. (2017). Troubled water under the bridge: screening of River Mur water reveals dominance of CTX-M harboring *Escherichia coli* and for the first time an environmental VIM-1 producer in Austria. Sci.Total Environ. 593, 399–405. 10.1016/j.scitotenv.2017.03.138 28351808

[B46] Zhang X. X. Zhang T. Fang H. H. (2009). Antibiotic resistance genes in water environment. Appl. Microbiol. Biotechnol. 82, 397–414. 10.1007/s00253-008-1829-z 19130050

